# Simulating Tsunami Inundation and Soil Response in a Large Centrifuge

**DOI:** 10.1038/s41598-019-47512-x

**Published:** 2019-07-31

**Authors:** M. Exton, S. Harry, B. Kutter, H. B. Mason, H. Yeh

**Affiliations:** 1 Oregon State University, School of Civil and Construction Engineering, Corvallis, OR 97331 USA; 2 University of California, Davis, Department of Civil and Environmental Engineering, University of California, Davis, CA 95616 USA

**Keywords:** Natural hazards, Civil engineering

## Abstract

Tsunamis are rare, extreme events and cause significant damage to coastal infrastructure, which is often exacerbated by soil instability surrounding the structures. Simulating tsunamis in a laboratory setting is important to further understand soil instability induced by tsunami inundation processes. Laboratory simulations are difficult because the scale of such processes is very large, hence dynamic similitude cannot be achieved for small-scale models in traditional water-wave-tank facilities. The ability to control the body force in a centrifuge environment considerably reduces the mismatch in dynamic similitude. We review dynamic similitudes under a centrifuge condition for a fluid domain and a soil domain. A novel centrifuge apparatus specifically designed for exploring the physics of a tsunami-like flow on a soil bed is used to perform experiments. The present 1:40 model represents the equivalent geometric scale of a prototype soil field of 9.6 m deep, 21 m long, and 14.6 m wide. A laboratory facility capable of creating such conditions under the normal gravitational condition does not exist. With the use of a centrifuge, we are now able to simulate and measure tsunami-like loading with sufficiently high water pressure and flow velocities. The pressures and flow velocities in the model are identical to those of the prototype yielding realistic conditions of flow-soil interaction.

## Introduction

**Figure 1:**
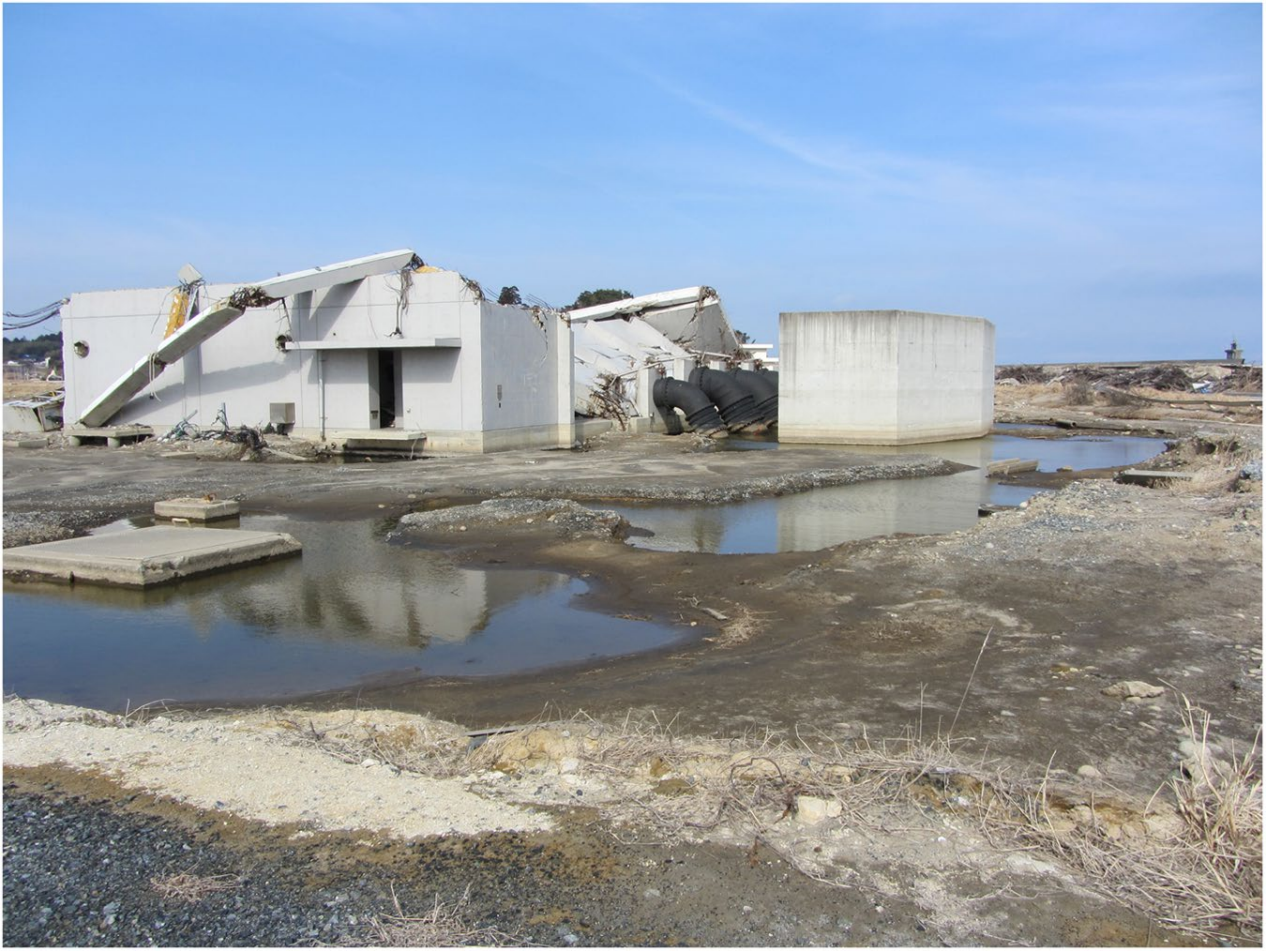
A scene following the 2011 Heisei Tsunami in Idagawa, Fukushima, Japan, showing destruction of a reinforced-concrete structure and severe scour surrounding the structure.

Fast surging flows on land accompanied by tsunami inundation affect the underlying soil medium and can result in severe scour or potentially liquefaction^1,2^. Figure 1 shows a photograph of a post-tsunami condition after the 2011 Heisei Tsunami in Japan. The powerful tsunami flow caused scour around the foundation and destroyed this reinforced concrete structure. The rarity of tsunamis makes them difficult to study in the field. The flow is transient and highly turbulent; studying soil response during tsunami loading is challenging. Realistic experimental data are needed to further understanding of the effects of tsunamis on soil. Understanding soil response during tsunami inundation is difficult using traditional water-wave tanks owing to unavoidable scale effects. Here, we use a novel laboratory apparatus designed to be operated on a large centrifuge, which controls the body force of the model. The apparatus enables the exploration of soil response to the tsunami-like inundation flows associated with both the flooding and drawdown processes.

Tsunamis differ from storm-surge inundation and river flows in both their temporal and spatial scales. Generally, river flooding occurs in several hours or more, and wave action associated with storm surge occurs with a period of 20 seconds or less. Tsunamis have an intermediate time scale, with an inundation time on the order of several to tens of minutes. Wind-generated waves have back-and-forth oscillatory motion, river flows do not change in direction (from upstream to downstream), and tsunamis have runup in one direction and drawdown in the opposite direction. For example, tsunami inundation observed in Kesen-numa, Japan, during the 2011 Heisei Tsunami showed the entire inundation process for the first wave lasted approximately 25 minutes^3^. The runup speed was approximately 3 m/s with a flow depth of approximately 4 m and the drawdown speed was approximately 6 m/s (with the maximum speed of 11 m/s)^3^. Tsunamis’ spatiotemporal scales are the reason that scaled-down laboratory experiments to study tsunami effects are difficult. The foregoing flow characteristics yield a Reynolds number on the order of 1 × 10^7^ ~ 2 × 10^7^ and a Froude number of approximately 0.5 ~ 1.0. A large Reynolds number causes substantial mismatch with the corresponding scaled-down laboratory simulations, and the mismatch makes the outcomes from the experiments unreliable. Suppose we use a 1/40 scaled-down model, which is possible in a laboratory water tank. Then, to match the Froude number, the Reynolds number would be 5 × 10^4^ ~ 1 × 10^5^ which is mismatched by 2 to 3 orders of magnitude. Additional scaling relationships are necessary in a scaled-down model when soil is involved to achieve the dynamic similitude in the soil domain.

**Figure 2:**
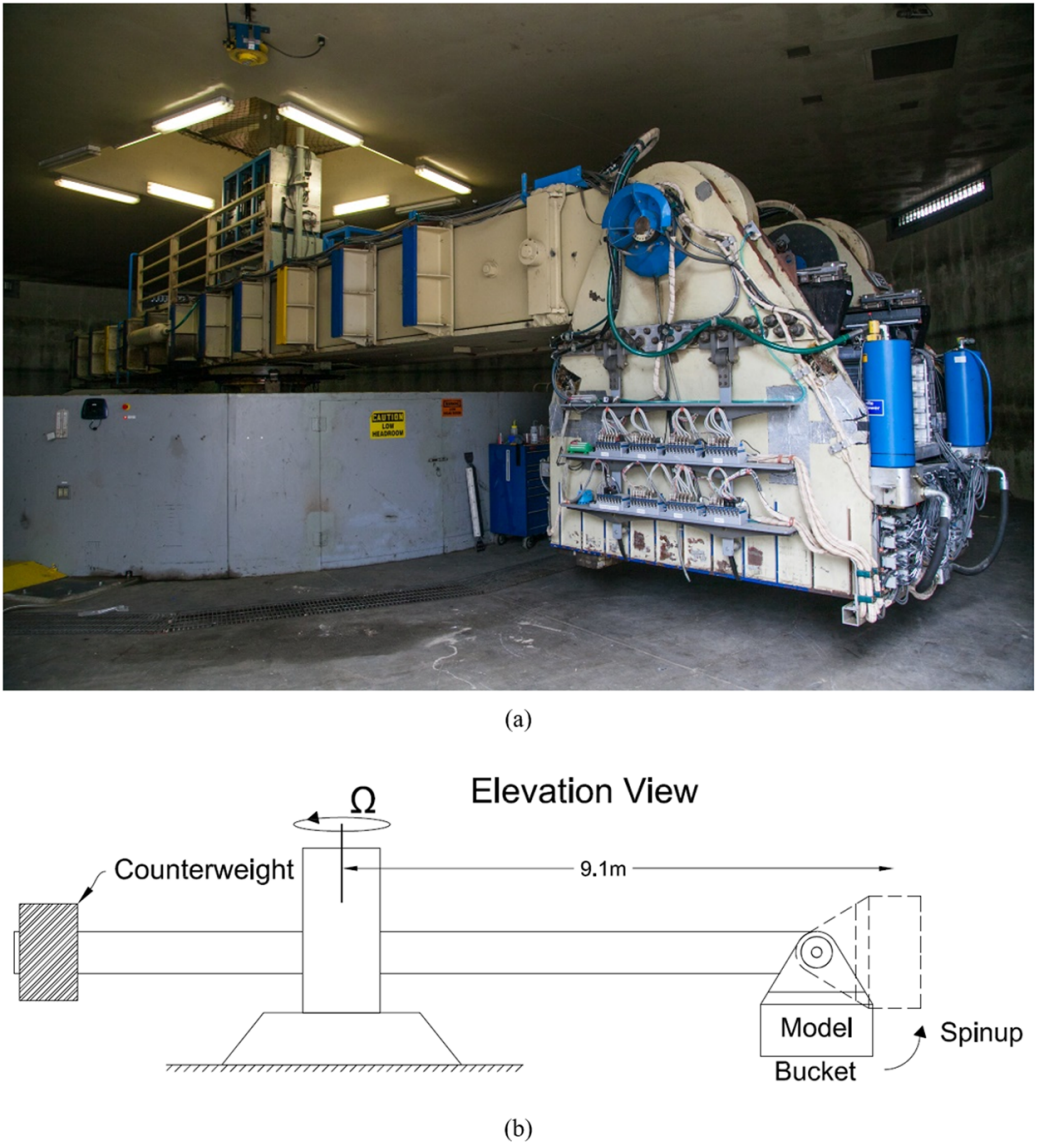
(**a**) A photograph and (**b**) a schematic of the geotechnical centrifuge at the Center for Geotechnical Modeling, University of California, Davis (photo courtesy of Dr. Dan Wilson).

To reduce the excessive scale effects, we use a novel laboratory apparatus on a centrifuge. A picture and schematic of a centrifuge is presented in Fig. 2. Centrifuge facilities are regularly used for studies of geotechnical engineering problems, but with no surface fluid flow involved. In the past, there have been a few attempts to use a centrifuge for water-flow problems. Sassa and Sekiguchi^4^ studied soil response under progressive water-wave action using centrifuge modeling. Other previous works^5–8^ were primarily aimed at demonstrating the performance of coastal structures during the runup stage of tsunami inundation.

The body force within a centrifuge model is increased by controlling the centripetal acceleration on the model, which is the primary advantage of using a centrifuge apparatus to investigate soil response. The experimental results are converted from a model scale (decreased length scale and increased body force) to a prototype scale under Earth’s gravity. For our experiments, there are two primary domains of concern: the fluid-flow domain and the soil domain. In addition, the air-water interface and the water-soil interface are important. Because the model experiences centrifugal forces created with a finite radius of rotation, the Coriolis effect could also influence the experimental results.

Our apparatus is designed to realistically model tsunami runup and drawdown processes and to yield quantitative measurements of the flow velocities, depths, and pressures. The apparatus and the sophisticated data acquisition system on the centrifuge allows us to make measurements of soil response; in particular, we measure the pore water pressure during tsunami loading. Here we demonstrate and discuss: 1) flow patterns of the initial surge front onto a quiescent water body, 2) flow velocity and pressure measurements of the initial surge front onto the soil medium, and 3) pressure transmission through the soil medium. The results of the experiments will be presented, focusing on the physical processes of tsunami-soil interaction, how they are represented in the centrifuge environment, and how they compare to the target prototype conditions.

## Results

Before examining the results, we note typical conditions of a large and realistic tsunami. For significant tsunami events (e.g. see NGDC/WDS Global Historical Tsunami Database^9^), the maximum flow depth ranges between 4 to 8 m and the maximum flow speed ranges between 5 to 10 m/s. Based on video footage from many sources taken during the 2004 Indian Ocean Tsunami and the 2011 Heisei Tsunami events, depths of the leading flooding flows are anticipated between 1 to 2 m.

**Figure 3:**
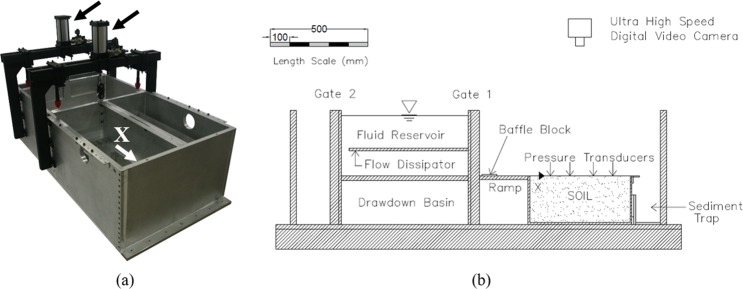
(**a**) A photo of the apparatus. Arrows indicate the pneumatic actuators that open the gates; (**b**) a plan view schematic drawing of the apparatus. Dimensions are in millimetres. Gate 1 opens to initiate the flooding stage followed by Gate 2 for the drawdown stage. No gate opening device is shown in the sketches for brevity but it can be seen in the photo.

To create the flooding flow in our centrifuge experiments, the fluid impounded in the reservoir is released by rapidly lifting a gate with the aid of a pneumatic actuator (analogous to a sudden dam break). After the specimen is flooded, the water is withdrawn by lifting another gate. With the use of two gates, the apparatus is capable of mimicking the flooding and drawdown processes in a controlled manner. Schematic views and a photo of the apparatus are shown in Fig. 3.

### Flow results in the centrifuge model

Here we describe the fluid flows created to simulate tsunami-like inundations. To simulate fluid flows in a scale down model, geometric, kinematic, and dynamic similitudes must be satisfied in principle. Geometric and kinematic similitudes can be achieved by constructing an undistorted model of the corresponding prototype. Dynamic similitude is achieved if the ratios of dynamic components of the model, such as the inertial force to the surface force and the inertial force to the body force, match those of the prototype. In a Newtonian fluid environment, the dynamic-component ratios arise explicitly in the normalised equation of motion, i.e. Navier-Stokes equation:

1 $$S\frac{\partial \overrightarrow{u}}{\partial t}+\overrightarrow{u}\cdot \nabla \overrightarrow{u}=-\frac{1}{E}\nabla p+\frac{1}{R}{\nabla }^{2}\overrightarrow{u}+\frac{1}{{F}^{2}}\overrightarrow{k},$$

where all of the parameters are scaled to the order unity with appropriate representative values and the magnitude of each term is represented by the coefficient. In (1), $\overrightarrow{u}$ is fluid velocity, *p* is pressure, *t* is time, $\overrightarrow{k}$ is unit vector pointing in the direction of the body force, and ∇ is the del operator. The non-dimensional coefficients in (1) are:

2 $$\begin{array}{cc}{\rm{Strouhal}}\,{\rm{number}}:S=\frac{{L}_{0}}{{u}_{0}{t}_{0}}; & {\rm{Euler}}\,{\rm{number}}:E=\frac{{\rho }_{0}{u}_{0}^{2}}{{p}_{0}};\\ {\rm{Reynolds}}\,{\rm{number}}:R=\frac{{\rho }_{0}{u}_{0}{L}_{0}}{{\mu }_{0}}; & {\rm{Froude}}\,{\rm{number}}:F=\frac{{u}_{0}}{\sqrt{{g}_{0}{L}_{0}}};\end{array}$$

where *L*_0_, *t*_0_, *u*_0_, and *p*_0_ are representative scales of the length, time, velocity, and pressure, respectively, and *ρ*_0_, μ_0_, and *g*_0_ are the fluid density, viscosity and the body force per unit mass, respectively. The coefficients in (2) represent force ratios: *S*, local inertial force to advective inertial force; *E*, inertial-to-pressure forces; *R*, inertial-to-viscous forces; *F*, square root of inertial-to-gravitational forces. Perfect dynamic similitude is difficult to achieve in practice, because either Reynolds or Froude number is mismatched unless the body force and the fluid density or viscosity can be controlled. As will be discussed later, dynamic similitudes of the boundary conditions are also important: viz. the air-water interface, as well as water-soil interface.

Dynamics associated with tsunami inundation processes are dominated by the gravitational force; hence, it is a common practice to match the Froude number of a model with that of a corresponding prototype, which is referred to as the Froude law. With a centrifuge facility, the body force of a model, *g*_0_, can be adjusted by the centripetal acceleration, often expressed by a multiple of Earth’s gravitational acceleration, say *Ng*, where *g* is the gravitational acceleration. In this study, we set the centrifuge to create *N* = 40. If we match the geometric similitude with all lengths scaled down by a factor 1/*M*, then to maintain the Froude law, the velocity scale in the model should be (*N*/*M*)^1/2^. Consequently, the time scale of the model should be 1/(*MN*)^1/2^ of that of prototype. In conventional geotechnical centrifuge modeling^10,11^, the geometric model scale is set to be 1/*N* by taking *M* = *N*. With this scaling convention, the model velocity becomes the same as that of the prototype, resulting in equal pressures and equal stresses in model and prototype. Identical stresses are important to capture the effects on the boundaries. Hereinafter, we discuss the scaling of the centrifuge experiments with *M* = *N*.

Under the Froude law, if the same fluid (i.e., water) were used in a centrifuge model as in the prototype, the mismatch in Reynolds number would be 1/*N*, which is better than the mismatch for a model tested in a 1 g environment, 1/*M*^3/2^. In other words, the mismatch in Reynolds number in the centrifuge is reduced under Froude law by the square root of the length scale. If turbulent flow cannot be fully established in a 1 g scale model, the centrifuge may be helpful in increasing the Reynolds number to the fully turbulent regime.

Due to mechanical constraints (see Fig. 2), centrifuge models are necessarily small (i.e., less than 2 m) compared to full-scale wave flume models; thus, it is feasible to use a fluid with a different viscosity than water^11^. Then, the Reynolds number and the Froude number can be matched between the prototype and the centrifuge model: a fluid with 1/*N* viscosity ensures that all of the dimensionless numbers in (2) are matched. Using a 1/*N* viscous fluid is theoretically possible, but finding such a fluid is difficult. Nonetheless, as we discussed earlier, even if the same fluid were used in a centrifuge, the mismatch in Reynolds number would be better than the mismatch under a 1 g environment.

**Figure 4:**
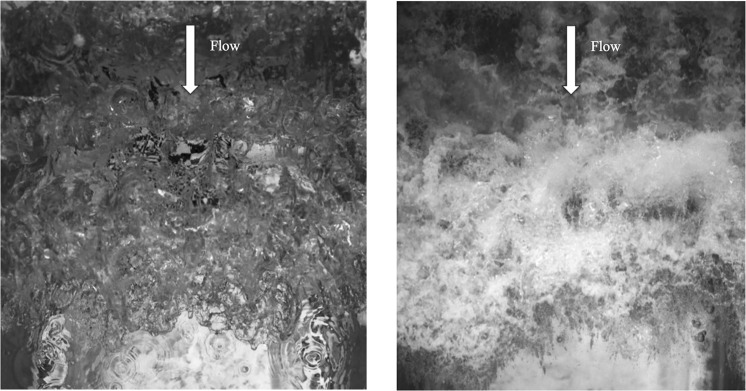
Surge front generated from the centrifuge apparatus: (**a**) under 1-g condition, and (**b**) under 40-g condition. Flow is from the top to the bottom of the images. Several streaks shown in (**b**) are the wake formations from the baffles installed on the flow ramp (see Fig. 3). Also, entrapped bubbles created by the impingement are detected in (**b**). On the other hand, a few droplets in front of the surge front can be seen in (**a**). No such droplets are detected in (**b**) because the large body force (40 g) disallows any droplet to fly high.

Figure 4 shows the comparison of the flooding flows over an initially quiescent water body (no soil is placed in the specimen box – this situation is similar to when a tsunami’s overland flow enters a water body such as an estuary or a lake): (a) shows the process under Earth’s gravity (1 g model), and (b) shows the process under the enhanced body force on the centrifuge operated with *N* = 40 (40 g). The flow depth is controlled by the physical configurations of the apparatus; hence, the flow depth within the 1 g and 40 g models are comparable: the measured flow depth of the leading surge is 0.047 m in the 1 g model and 0.030 m in the 40 g model. Since the viscous boundary-layer thickness is inversely proportional to the square root of the flow speed, we conjecture that the slight difference in flow depth is due to the development of the boundary-layer. The flow velocity in the 40 g model in Fig. 4 is theoretically *N*^1/2^ (=40^1/2^) times greater than that of the 1 g model. In fact, the measured speed of the leading head of the flow is 1.75 m/s for the 1 g model and 10.5 m/s for the 40 g model; hence, the flow speed is close to the predicted value (=1.75 × 40^1/2^ = 11.1 m/s). The resulting Reynolds number, *R*, for the 1 *g* model is 8.2 × 10^4^, and 3.2 × 10^5^ for the 40 *g* model. The Froude number for the 1 g model is 2.6 and 3.1 for the 40 g model. The flow conditions demonstrate the substantial reduction in the mismatch of values for the Reynolds number in the 40 g centrifuge model.

Figure 4 exhibits that breakup bubbles/droplets are formed in the 40 g model, while the small features of the surface pattern in the 1 g model is relatively smooth. This is a consequence of the scaling at the air-water interface. Non-dimensionalising the jump condition of conservation of momentum at the stress-free air-water interface results in the Weber number, which describes surface-tension effects^12^. The Weber number, *W*, can be expressed as:

3 $${\rm{Weber}}\,{\rm{number}}:W=\frac{{\rho }_{0}{L}_{0}{u}_{0}^{2}}{{\gamma }_{0}},$$

where *γ*_0_ is the surface tension. The difference in surface pattern in Fig. 4 is due to the mismatch in the Weber number. Under the Froude law the mismatch is 1/*N*^2^ for the 1 g model, whereas it is 1/*N* for the 40 g model. Hence the surface tension effect is much stronger in the 1 g model than the prototype and the 40 g model; the strong surface tension prevents the air-water interface from breaking into bubbles and droplets. Bubble and droplet formations appear more realistic in 40 g model in Fig. 4 than those in 1 g model. A summary of the flow characteristics for the conditions shown in Fig. 4 is presented in Table 1.

**Table 1:** Flow characteristics for the targeted prototype, the 1 g model and the 40 g model. Here, the flow depth is used for the length scale computed for Reynolds number, Froude number, and Weber number.

	Target prototype	1 g model	40 g model
Flow speed (m/s)	5~10	1.75	10.5
Flow depth (m)	1~2	0.047	0.030
Reynolds number	5 × 10^6^~2 × 10^7^	8.2 × 10^4^	3.2 × 10^5^
Froude number	1.1~3.2	2.6	3.1
Weber number	3 × 10^5^~3 × 10^6^	2.0 × 10^3^	4.6 × 10^4^

### Soil response of the centrifuge model

**Figure 5:**
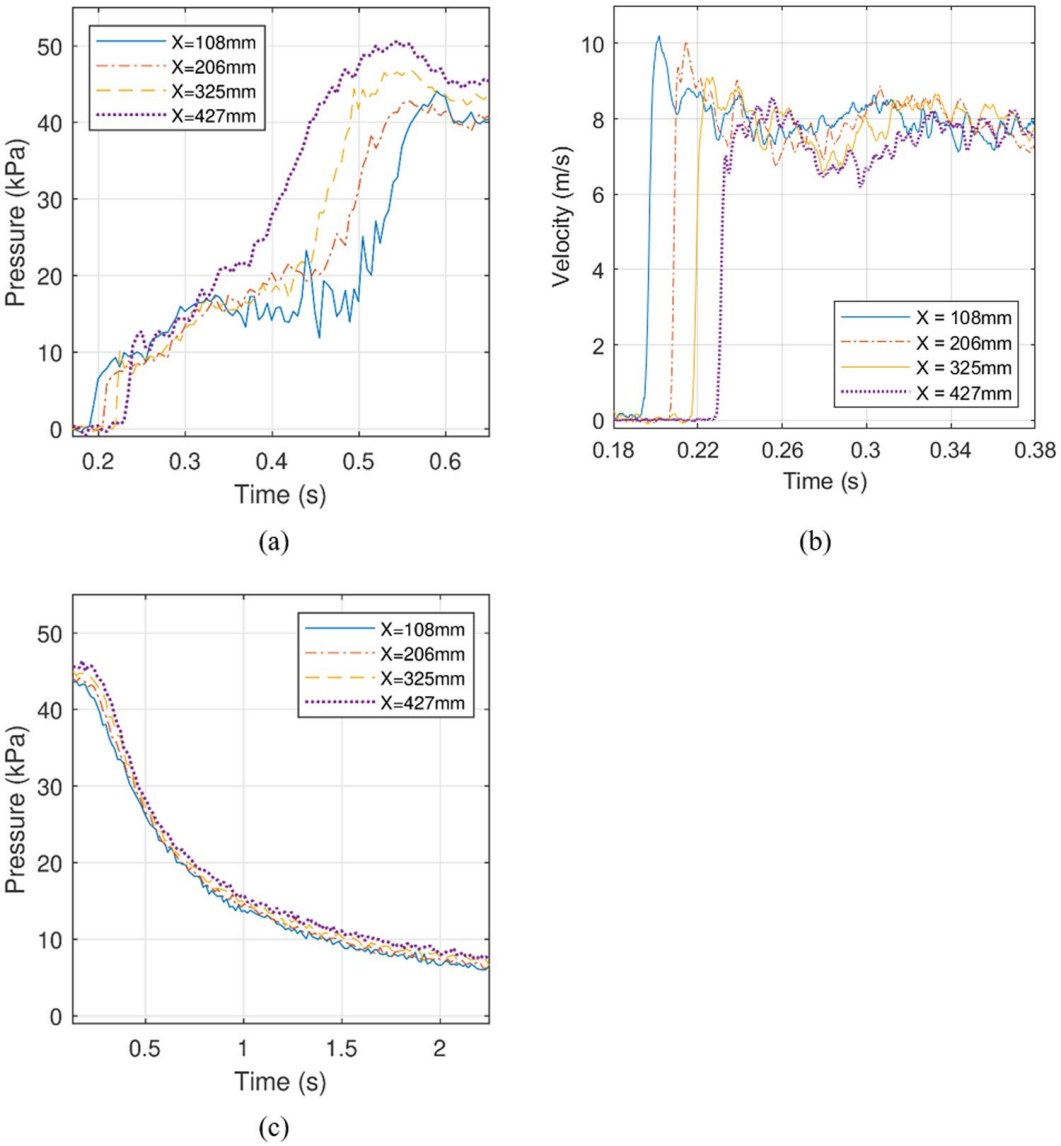
Quantitative flow measurements: (**a**) water pressures at the soil bed, (**b**) flow velocities during the flooding stage, and (**c**) water pressures at the soil bed during drawdown stage. The measurements were made at the location X from the initial contact with the soil surface (see Fig. 3). The leading front of the flooding flow has a velocity of 8 to 10 m/s with a flow depth of about 2 cm. The total drawdown time is 3 seconds and the mean flow speed is approximately 1.2 m/s. Note that the time for each stage begins when the reservoir discharge gates open. Four minutes elapsed between stages. ((**a**) and (**c**) are based on the data exhibited in Exton *et al*.^20^).

**Figure 6:**
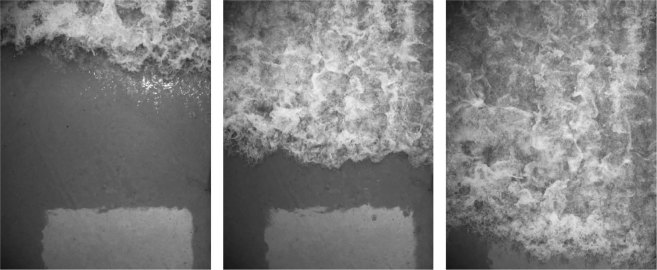
The discharging flow onto the soil is captured with the high-speed video camera. The footage was taken at 4000 frames per second with an exposure time of 1/10000 seconds and a resolution of 1024 × 1024; the time interval between these photos is 16 ms. Using this footage, the flow velocities are computed with the optical flow algorithm^24^. (The frames are based on the experiments by Exton *et al*.^20^).

Figure 5a shows the flooding flow conditions over the soil strata in the 40 g centrifuge model measured using pressure transducers arranged parallel to the direction of flow on the surface of the specimen box at the edge of the soil model (see Fig. 2 for locations). Figure 5b shows the velocity time series at four locations along the centreline of the soil model corresponding to the pressure transducer locations (obtained using the optical flow algorithm as discussed in “Methods.”). The leading front of the runup flow has a velocity of about 8 to 10 m/s with a flow depth of about 2 cm. Figure 5c shows the achieved drawdown flow conditions. The total drawdown time is 3 seconds and the mean flow speed, determined by particle tracking, is 1.2 m/s. Figure 6 shows three frames captured using the high-speed camera during the flooding stage. The flow speed at these locations is 8.5 m/s, and the maximum flow depth is 11.5 cm. Based on the Froude law, the corresponding flow depth of the prototype would be 4.6 m with the same flow speed (8.5 m/s). Therefore, the model flow condition represents a realistic tsunami event as described earlier.

Unlike fluids, there is no established theory for soil dynamics because soils are a multi-phase particulate medium, and the continuum hypothesis is violated. Consequently, soil dynamics are modeled by semi-empirical theories. For example, Terzaghi^13^ introduced a linear, parabolic partial differential equation, which is used to describe the dissipation of pore-water pressure through soil pores. Terzaghi’s equation is extended to obtain the consolidation model of Biot^14^, which describes the dynamics of statically coupled pore fluid and soil. Furthermore, Biot^15^ extended the preceding models further to incorporate inertial effects of soil grains. The Terzaghi equation is the basis of the more complex consolidation theories, so we use it to describe dynamics of fully saturated soil mediums. Non-dimensionalising the Terzaghi equation yields:

4 $$\frac{\partial {p}_{e}}{\partial t}=T{\nabla }^{2}{p}_{e},$$

where *p*_*e*_ is the pore-water pressure in excess over the hydrostatic condition, and *T* is termed the Terzaghi number (also known as the non-dimensional time factor^16^ in the geotechnical engineering community):

5 $$T=\frac{{c}_{v}{t}_{0}}{{L}_{0}^{2}},$$

where *c*_*v*_ is the consolidation coefficient that can be expressed by:

6 $${c}_{v}=\frac{k}{{m}_{v}{\gamma }_{w}}=\frac{\kappa }{{\mu }_{0}}\frac{{E}_{v}}{1-{\varphi }_{0}},$$

where *k* is the hydraulic conductivity, *m*_v_ is the coefficient of volume change, and *γ*_*w*_ is the unit weight of water^17^. Alternatively, we could express *c*_*v*_ with the intrinsic permeability, *κ*, the fluid viscosity, *µ*_0_, the porosity, *ϕ*_0_, and the bulk modulus of elasticity, *E*_*v*_. The simplest and necessary requirement for dynamic similitude in the soil domain is to match the Terzaghi number *T*. Note that the intrinsic permeability can be estimated by Kozeny’s equation^18^ in terms of sediment grain diameter *d*_0_:

7 $$\kappa ={f}_{1}(s){f}_{2}(\varphi ){d}_{0}^{2},$$

where *f*_1_(*s*) is the shape factor and *f*_2_(*ϕ*) is the porosity factor^18^. If we assume no difference in the soil structure between the model and prototype scales, then $\kappa \propto {d}_{0}^{2}$; therefore, the Terzaghi number is proportional to *d*_0_^2^*t*_0_/(*μ*_0_*L*_0_^2^) and is mismatched by *T*_*m*_/*T*_*p*_ = 1/*N* when an identical fluid is used for the model and prototype (the subscripts *m* and *p* represent quantities in the model and the prototype, respectively), Here, we consider the soil particles used in the centrifuge to be 1/*N* times the size of the prototype particle size, although scaling down the soil particle size may create problems in soil response (see §3). Accordingly, it is theoretically possible to match the Terzaghi number for the model and the prototype if we use a fluid with smaller viscosity than that of the prototype: *μ*_*m*_/*μ*_*p*_ = 1/*N*, although finding such a fluid is impractical.

The boundary condition at the fluid-soil interface is important for soil response. At the water-soil interface, the Shields number describes the soil stability (i.e., the relationship between the shear force on the soil and the net body force), and the Shields number, Θ, can be expressed as

8 $${\rm{Shields}}\,{\rm{number}}:\Theta =\frac{{\tau }_{0}}{{\rho }_{0}{g}_{0}{d}_{0}({S}_{g}-1)},$$

where *τ*_0_ is the bed shear stress, and *S*_*g*_ is the specific gravity of the sediment particles. For fully turbulent, steady flows, the bed shear stress, *τ*_0_, is proportional to *ρ*_0_ *u*^2^. Then, the Shields number matches under the Froude-law similitudes.

**Figure 7:**
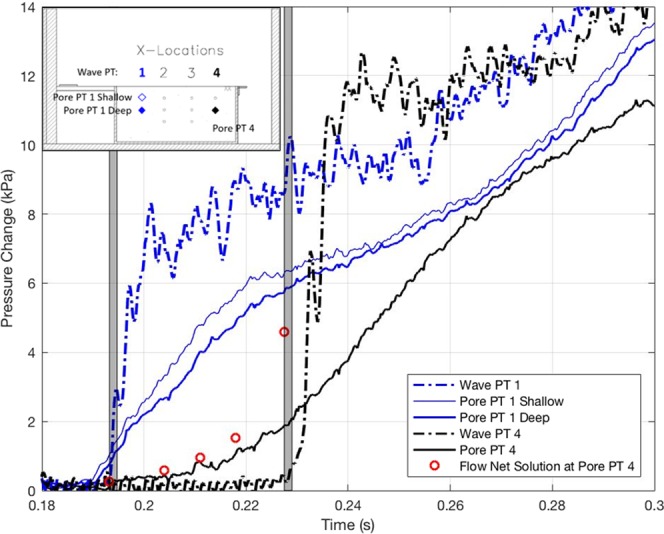
Temporal variations of pressures at Location 1 (*X* = 108 mm) and Location 4 (*X* = 427 mm) during the flooding stage. Solid lines show the measured pore-water pressures (Pore PT) in the soil; broken lines, the water pressures at the soil surface (Wave PT); shaded vertical columns represent the arrival time of the surge tip at Locations 1 and 4 determined from the high-speed video footage; hollow circles show the temporal variation of the pore-water pressures at Location 4 that were computed based on the quasi-static assumption.

For the 40 g centrifuge model with flow onto a soil specimen model, Fig. 7 shows the response of 1) the first and fourth pressure transducers to encounter the model wave during runup at *X* = 108 and 427 mm on the soil surface along the side wall (note that the origin of *X*-coordinate is set at the upstream edge of the soil specimen box), 2) the arrival times of the leading edge of the surge detected by the high-speed video images, and 3) pore-water pressures at three embedded locations. The data show that the arrival times detected by the surface pressure transducers are consistent with the timings detected from the high-speed video. At Location 1 (*X* = 108 mm), the shallow pore-water-pressure gage responds slightly faster than the deeper gage, as anticipated for a transient process. In addition, at Location 1, the pore-water pressure gages’ response time is slightly earlier (≈ 0.002 s) than the arrival time of the surge detected by the pressure transducer and the video images.

The miniscule discrepancy of arrival times in Fig. 7 could be explained either by a realistic physical soil response, or as error caused by the difference in the sensor locations; more specifically, the surface pressure transducer measurements and the video detection were made along the sidewall, whereas the pore-water pressures were measured in the sediments along the centreline of the sediment container. Nonetheless, the foregoing explanation cannot be applied to explain the large discrepancy in pressure arrival times at Location 4 (*X* = 427 mm).

**Figure 8:**
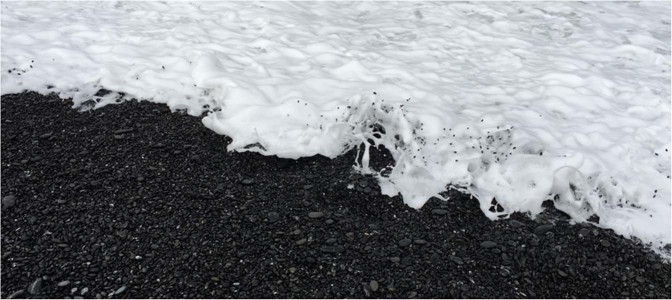
Northern California: the beach is made of coarse sand particles and the slope is steep. The sand particles pop up immediately before the arrival of a surge front.

The large discrepancy observed in Fig. 7 between the surge arrival time recorded by the pressure transducers at the surface of the model and the pore-pressure transducer embedded in the model at Location 4 can be explained by the difference in the pressure transmission speed between the free-surface flows and the semi-confined soil medium. Water-wave pressure propagates with the deformation of the water surface. On the other hand, the pressure within the fully saturated soil can transmit faster than the free-surface surge motion and is controlled by the speed of a compressional wave in the medium. If we assume the saturated soil medium to be a rigid porous body with an infinite speed of pressure transmission (just as an incompressible fluid in a rigid closed conduit), then the pressure pattern within such a hypothetical soil medium would be equivalent to the state described by a flow net (computed by an elliptic, time-independent partial differential equation). In reality, the pressure transmission speed is finite; hence the flow net solution is the extreme case. Nonetheless, the flow net solutions are computed for five boundary conditions, assuming complete saturation, isotropy and homogeneity of the soil specimen: the front of the uniform surge with the pressure 10 kPa being at five locations: *X* = 108, 206, 325, 427 mm (the same locations of the pressure sensors), plus the midpoint of the sand specimen (*X* = 265 mm). The predicted pressures at Location 4 (*X* = 427 mm) are computed at each instance of the surge front locations, and the results are plotted with the circle marks in Fig. 7. The results provide an explanation for the observed early response in pore-water pressure within the soil. The close agreement of the measurements with the flow net prediction implies that the pressure transmission speed is fast, or equivalently the consolidation coefficient in the Terzaghi equation (Eq. 5) is large enough for this phenomenon in the model. As stated earlier, the model soil is a fine sand with a mean particle diameter of 0.21 mm. Therefore, if we use the same length scale for the grains (micro) as we do for the soil model (macro), the corresponding sediment grain diameter in the prototype is 8.4 mm, which can be categorised as pebbles (or fine to medium gravels). If much finer sand grains were used in the model, we conjecture that the pressure propagation is anticipated to be slower due to a finite value of the speed of sound in the sand/water mixture, and the process may more closely follow the diffusion process modeled by (4). Incidentally, a possible result of the upward seepage just ahead of the wetting front can be observed in a real beach with relatively small pebbles as shown in Fig. 8. During surges on a relatively steep beach face, if the pore-water pressure in the soil increases ahead of the leading tongue of the surge and surpasses the weight of pebble grain, pebbles can pop up ahead of the leading tongue of the surge.

### Effect of centrifuge rotation

Because the radius of centrifuge rotation is finite, any motion in the radial and the tangential directions alter the angular momentum; consequently, the flow induces the deflective motion to its “right” (cum sole), which is the Coriolis effect. To estimate the importance of the Coriolis effect, we use the Rossby number, *R*_0_, which is a dimensionless number often used in the field of oceanography,

9 $${R}_{0}=\frac{{u}_{0}}{2{\rm{\Omega }}{L}_{0}},$$

where Ω is the angular speed. The Rossby number represents the ratio of the inertial force to the Coriolis effect. If the Rossby number is ‘small’, then the Coriolis effect is important.

We calculate the Rossby number, *R*_0_, for the initial runup flow to be approximately 5, which indicates that the Coriolis effect is weakly important. Although the Coriolis effect becomes more important when the flow speed decreases and the flow depth increases (e.g., during drawdown), the Coriolis effect is limited to the flow in the lateral (tangential) and vertical (radial) flow directions. Accordingly, the primary flow direction in our experiments should be unaffected by the Coriolis effect.

## Discussion

We introduce a unique experimental apparatus used in the centrifuge devise to study hydrodynamics and soil response under tsunami-like inundation processes. Controlling the body force (i.e. centrifugal force) can mitigate the scale effects for the Froude law by increasing the Reynolds number by *N*^1/2^ times the condition of the 1 g environment. This can be a significant advantage for the centrifuge, because a sufficiently large Reynolds number means potentially creating fully turbulent flows in which viscous effects become unimportant, hence the flow becomes independent of the Reynolds number. Another important advantage of use of centrifuge is to enable us to realise the identical hydrodynamic condition on the soil-fluid interface as the prototype. Note that identical stresses on the soil surface are important, because soil strength properties such as friction angle, dilatancy, and elastic moduli are nonlinear functions of stress^16^. This requirement can be fulfilled in centrifuge experiments, but not standard 1 g experiment. This is because the velocity and pressure of the scale-down model in a centrifuge environment are the same as those of the prototype (when the geometric scale *M* = *N*).

Because the centrifuge requires the mechanical device to spin, the size of the apparatus is necessarily limited and small. The smallness of the specimen provides us with an opportunity to implement options that cannot be achieved in a traditional large-scale 1 g model; for example, the use of a fluid different than water to control viscous effects is possible, and the soil specimen with complete saturation can be achieved as will be discussed in “Methods”. The small physical size of the apparatus is, on the other hand, a drawback. First, the instruments must be very small to obtain data with adequate spatial resolution, and they must be durable under the enhanced forces induced by the large centripetal environment. Because the centrifuge time is *N* times faster than the prototype time, high-speed data samplings are essential; this includes video equipment. To avoid significant relative errors in experimental conditions, the setup of the experimental specimen must be precise and much more experimental care is required than for traditional 1 g experiments.

In our study, we consider the soil particle size is scaled by *N*. However, due to the behavior of soil as an assembly and the necessary interparticle contact forces, the grain size is not simply a length scale. According to Kutter^11^, interparticle contact forces depend on stress and the number of contacts per area, which depends on the absolute particle size, not on the scaled particle size and not on the body force. Soil strength, stiffness, dilatancy, and the porosity of soil have nonlinear dependence on stress in the soil. This is one of the reasons why most of the geotechnical engineering experiments in centrifuge do not treat the soil particles as being scaled: i.e. they consider that the soils in the prototype and the model to be identical. If we consider the sediment particle size in the model and the prototype to be the same, then, to match the Terzaghi number, the viscosity in the model should be *N* times more viscous than that in the corresponding prototype. Recall that, to satisfy the dynamic similitude in the fluid domain, the viscosity must be scaled by 1/*N* instead, which is a direct contradiction. As an aside, because a majority of geotechnical engineering problems modelled in the centrifuge have no fluid flow on the soil surface, a well-established practice in geotechnical centrifuge modeling is mixing water with hydroxypropyl methylcellulose or using silicon oil to increase the liquid viscosity without significantly increasing the fluid density^11^. Viscosity scaling is primarily used to achieve similitude relating to movement of the liquid in soil-pore spaces during a dynamic loading scenario (such as earthquakes)^19^. However, it has yet to be demonstrated if the foregoing practice (unscaled soil particle size), which is established in the geotechnical engineering field, is the best practice for the problems involving surface flows. It is also unclear if the above explanation for not scaling soil particle size is applicable to non-cohesive and relatively large granular materials. Regardless, the treatment for soil particle size remains a point of future research, especially when surface flows are considered.

In summary, flows of tsunami inundation are simulated in a novel apparatus in the large centrifuge facility at the University of California, Davis (discussed in “Methods”). Increasing the body force by centripetal acceleration creates free-surface flows with a high-Reynolds number in a scaled down (1/40) model; the resulting flow pattern resembles reality closer than the result from the corresponding 1 g simulation. Soil responses to the fast-moving flow front are studied. The measurements show that the pore-water pressure in the soil propagates faster than the tsunami surge front. On one hand, if we consider that the soil medium is the extension of the water body, then the resulting pressure disturbance must propagate with the surge motion. On the other hand, if we assume the soil medium is rigid, then the pressure propagates with infinite speed if the fluid is incompressible; in this situation, the resulting pressure field can be computed by flow net: i.e., the pressure field can be modeled by an elliptic equation with no time dependence. Our data show that the propagation speed is fast but finite, suggesting that the phenomenon may be modeled by a combination of hyperbolic and parabolic equations.

In addition to the simulation results, we also carefully review and discuss the scale effects and interpretations of the simulated results for the corresponding prototype situations. Advantages and disadvantages of centrifuge experiments involved in free-surface flows and soils are discussed. Many details of centrifuge experiments need to be further explored, though compromises in scaling are an experimental reality for all physical models.

## Methods

### Experimental apparatus

To simulate a typical tsunami inundation flow, a specialised experimental apparatus was designed and constructed specifically for use in the geotechnical centrifuge at the Center for Geotechnical Modeling (CGM) at the University of California, Davis. The CGM centrifuge has a 9.1 m rotation arm, is capable of spinning to create 75 g of centrifugal force and has a payload capacity of 4500 kg (see Fig. 2). This large capacity allows sufficient space for the tsunami apparatus to be mounted on a swing platform at the end of the arm.

The tsunami apparatus is 1.93 m long, 0.94 m wide and 0.58 m deep: see Fig. 3. The size is small for hydraulic experiments under the normal gravitational force. Nonetheless, under 40 g of the centripetal acceleration, the size is equivalent to 77.2 m long, 37.6 m wide, and 23.2 m deep: it represents a huge laboratory tank. The soil model installed in the container (0.533 m long, 0.364 m wide, 0.25 m deep) represents the prototype soil field of 21 m long, 14.6 m wide and 9.5 m deep. For conservatism, we designed the apparatus to withstand the full design load condition (i.e. water filled to the rim of the tub) under 75 g of the centripetal acceleration. Therefore, the outside walls of the tub are made of 31.75 mm thick aluminum plates, the reservoir baseplate is 25.4 mm thick aluminum, and the gate-support walls are 44.45 mm thick aluminum plates (see Fig. 3 for a photograph of the apparatus). To supply a sufficient force to lift the discharge gates under the increased body force in the centrifuge, the pneumatic actuators have a 101.6 mm bore diameter and are operated with the air pressure of 1,000 kPa, yielding 8.8 kN of force. To regulate the fluid discharge, a horizontal plate with a narrow opening along the withdrawal gate (Flow Dissipater shown in Fig. 3) is installed. The flow is also directed with the aid of the vanes installed on the reservoir floor and the discharge ramp; the flow vanes reregulate flow as well as reduce the Coriolis effects caused by the downward flow (in the direction of the centrifugal force). Controlling the flow discharge with the dissipater plate is sufficiently effective and structurally simpler than a more sophisticated control device (e.g., Mariotte tanks used by Sassa *et al*.^6^). Immediately outside of Gate 1 (see Fig. 3), ramps with small baffle blocks were installed to allow re-regulation of the flooding flow onto the soil specimens; note that during the first moment of gate opening, the reservoir head is highest and gate opening is smallest; hence, dissipation is needed for the resulting thin, high-speed flow. More information about the apparatus is given by Exton *et al*.^20^.

### Model preparation

Fine-grained sand (identified as Ottawa F65 produced by US Silica, Ottawa, Illinois) is used for the experiments. The mean grain-size diameter is 0.21 mm, the specific gravity is 2.65, and the porosity is 0.38 (a relative density of 55%)^21^. A homogeneous sand specimen is created by releasing sand from a hopper in a consistent and systematic manner; namely, the sand particles are dropped at a specified height and at a specified pluviation intensity that is controlled by the size of the screen opening on the hopper. We calibrated the drop height and pluviation intensity to achieve the desired density. Once the model is built, it is then saturated with water. Care must be taken to create saturation in soils, because small amounts of air bubbles (or even dissolved air) could alter the soil response significantly^22^. Saturation is achieved by first de-airing water in a supply tank by decreasing the tank pressure below atmospheric, but not past the vapor pressure. The soil model is placed in the saturation chamber (i.e. an air-tight container) where a vacuum is applied to the model to remove the air. The saturation chamber is then flushed with carbon dioxide because it is 50 times more soluble in water than nitrogen. This procedure is repeated twice. Note that the carbon-dioxide flush helps minimise the emergence of gas bubbles during the experiments. Once the foregoing preparation is completed, the de-aired fluid is introduced slowly in the soil specimen. After the specimen is completely inundated, the vacuum is slowly released, and the small amount of low-pressure gas in trapped voids compresses and dissolves in the de-aired fluid. Using the preceding procedure theoretically results in 100% saturation^23^.

The small specimen size in the centrifuge apparatus enables us to achieve the truly saturated sediment condition with the foregoing vacuum-and-CO_2_ treatment. Such a treatment is extremely formidable – if not impossible – in any large-scale experiments under the normal gravitational condition. Note that the experiments without such a saturation treatment would contain significant uncertainty in soil response due to the compressibility of entrapped gas.

### Instrumentation

To measure the varying water depth, an array of four pressure transducers is placed along the sidewall at the level of the soil surface: *X* = 108, 206, 325, and 427 mm from the upstream edge of the soil specimen box as shown in Fig. 3. The water depth is estimated with the assumption of a hydrostatic pressure field. We recognise that such an assumption may yield errors at the leading surge tongue during the flooding stage of the experiments when the flow is highly turbulent. At this stage, there is substantial vertical acceleration and the leading edge is not controlled to be uniform across the specimen box. Except the extreme leading tip of the surging flow, examination of the digital video (GoPro Hero 4) confirms the appropriateness of the hydrostatic assumption.

A high-speed digital video camera (Photron Mini AX100) is used to estimate the flow velocities. The high-speed camera is needed to analyse the flows in the centrifuge, because the model time is scaled *N* times faster than that of the prototype: *N* = 40 for the present experiments. The high-speed camera records the soil surface from above and captures images at the rate of 4000 frames per second (100 frames per second at the prototype time scale). Temporal synchronisation with the data acquisition system is established with a high-precision electronic trigger. Prior to performing quantitative image analyses, the captured images are rectified by correcting for radial lens distortion.

Flow velocities are estimated from video images of the opaque aerated flow near the initial stage of reservoir discharge when the entrapped air bubbles in the turbulentflow are sufficient to allow the application of an optical flow algorithm^24^. We use the optical flow algorithm developed by Farneback^25^, which estimates the translation of light intensity between images based on polynomial expansion. The algorithm fits a second-order polynomial locally in the sequential images, then, utilising the exact solution for the translation of second-order polynomials, estimates the translation. In contrast to particle tracking technique, which is sparse estimates of the flow velocity only at the location of tracers, the optical flow estimates are taken at every pixel location. In our experiments, the Farneback^25^ optical flow algorithm works well to extract the flow velocities when there are sufficient entrained air bubbles in the flow. The resulting flow velocities are verified by the particle-tracking algorithm: for particle tracking, flow tracers (unexpanded polystyrene beads) are introduced into the reservoir so that velocity estimates can be performed using the captured images.

To measure the soil response, pore-water pressure transducers are installed within the soil specimen at various locations to form arrays in the longitudinal direction. The pore-water pressure is measured by securing a ceramic porous stone between the sensor diaphragm and the surrounding soil. The sensors are specifically designed for geotechnical centrifuge model testing. The sensor is necessarily small (approximately 7 mm diameter and 11 mm length) and the wire is thin (approximately 1 mm diameter) for the physically small size of specimen used in the centrifuge. The sensors are installed during construction of the soil model and the porous stones are saturated with the model. Centrifuge experiments are now practical due to the recent advances in miniature sensors and high-speed video cameras: in comparison with the experiments under normal gravitational body force, a specimen size used in the centrifuge is relatively small (1/N of the prototype) and the duration is short (1/N).

Once the apparatus is loaded onto the centrifuge arm, the soil specimen is secured within the apparatus and all instrumentation is connected to the data acquisition system. To begin the experiment, the “spin-up” commences. During spin-up, the centrifuge arm begins to rotate gradually until the target rotation rate is reached. The target rotation is determined by calculating the desired centripetal acceleration of the soil model. Once at the target acceleration, the flooding stage is initiated, followed by drawdown, and the data are recorded. The angular velocity of the centrifuge is then reduced to zero, which is a stage referred to as “spin-down.” This procedure provides us with an opportunity to make additional calibrations of the sensors *in-situ* by using the varying body force; such *in-situ* sensor calibrations cannot be possible for the normal gravity condition.

## Data Availability

This research was funded under the NSF-supported Natural Hazards Engineering Research Infrastructure (NHERI). All data produced under NHERI awards must be published on the public repository, DesignSafe-CI. This data is available upon request to the corresponding author and will be uploaded to DesignSafe-CI soon.
